# Attending work with chronic pain is associated with higher levels of psychosocial stress

**DOI:** 10.1080/24740527.2021.1889925

**Published:** 2021-05-18

**Authors:** Greig Adams, Tim V. Salomons

**Affiliations:** a Department of Psychology and Clinical Language Sciences, University of Reading Harry Pitt Building, Reading, UK; b Department of Psychology, Queen’s University, Kingston, Ontario, Canada

**Keywords:** presenteeism, psychosocial stress, chronic pain, workplace, productivity, absenteeism

## Abstract

**Background and Aims**: Much is known about the impact of pain in terms of medical costs and missed work. Less is known about its associations when individuals are present for work. This study examines “presenteeism” by analyzing the psychosocial costs of pain in the workplace, using the 2015 European Working Conditions Survey (EWCS).

**Methods**: We conducted cross-sectional analysis of 2384 individuals with chronic pain and 2263 individuals without pain (matched by age and sex) using data from the 2015 EWCS. We compared groups in terms of the following psychosocial factors: supervisor support, job responsibility, team cohesion, discrimination, threats/abuse, job competency, job reward, sexual harassment, stress, and job security. The groups were also compared in terms of days lost due to illness.

**Results**: People with pain were 64% less likely to view their job as rewarding (odds ratio [OR] = 0.61; 95% confidence interval [CI], 0.57–0.65), 47% more likely to be subjected to threats/abuse in the workplace (OR = 0.68; 95% CI, 0.63–0.73), 30% more likely to report poor supervisor support (OR = 0.77; 95% CI, 0.73–0.82), and 28% more likely to perceive discrimination in the workplace (OR = 0.78; 95% CI, 0.71–0.85). People with pain missed approximately nine more days of work per year than respondents without pain.

**Conclusions**: Chronic pain was associated with lower vocational fulfillment and feelings of being ostracized in the workplace. These findings suggest that the presence of pain in the workplace goes well beyond lost productivity due to absenteeism.

## RÉSUMÉ

**Contexte et objectifs**: On en sait beaucoup sur l’impact de la douleur en termes de frais médicaux et absences au travail. On en sait moins sur ses associations lorsque des individus sont présents pour travailler. Cette étude examine le « présentéisme » en analysant les coûts psychosociaux de la douleur au travail, à l’aide de l’Enquête européenne sur les conditions de travail 2015 (EECT).

**Méthodes**: Nous avons effectué une analyse transversale de 2 384 personnes souffrant de douleur chronique et 2 263 personnes sans douleur (jumelés par âge et sexe) à l’aide des données de l’EECT 2015. Nous avons comparé les groupes en fonction des facteurs psychosociaux suivants : soutien du superviseur, responsabilité au travail, cohésion de l’équipe, discrimination, menaces ou abus, compétence professionnelle, valorisation professionnelle, harcèlement sexuel, stress et stabilité de l’emploi. Les groupes ont également été comparés en termes de jours perdus pour cause de maladie.

**Résultats**: Les personnes souffrant de douleur étaient 64 % moins susceptibles de considérer leur travail comme enrichissant (rapport de cotes [RC] = 0,61 ; intervalle de confiance [IC] à 95 %, 0,57-0,65), 47 % plus susceptibles d’être soumis à des menaces ou abus dans leur lieu de travail (RC = 0,68; IC à 95 %, 0,63-0,73), 30 % plus susceptibles de déclarer un faible soutien du superviseur (RC = 0,77; IC à 95 %, 0,73-0,82) et 28 % plus susceptibles de percevoir une discrimination sur le lieu de travail (RC = 0,78; IC à 95 %, 0,71-0,85). Les personnes souffrant de douleur ont manqué environ neuf jours de travail de plus par an que les répondants sans douleur.

**Conclusions**: La douleur chronique était associée à un épanouissement professionnel moindre et à un sentiment d’être ostracisé sur le lieu de travail. Ces résultats indiquent que la présence de douleur sur le lieu de travail va bien au-delà de la perte de productivité due à l’absentéisme.

## Introduction

The economic burden of chronic pain is huge, costing the United States an estimated $560 to $635 billion in 2010, due to a combination of medical costs and productivity lost.^1^ Absenteeism—lost productivity due to absences from work—is a well-documented cost of chronic pain.^2–4^ Less is known about presenteeism, whereby unhealthy employees are physically present at work but unable to perform at full capacity. Studies that do tally lost productivity costs due to presenteeism are primarily based on employees’ self-reported productivity or approximate valuation methods that are difficult to verify.^5,6^ Assessing impact also involves understanding the different ways in which individuals with pain might struggle while at work.

Chronic pain can be invisible to employers, yet its psychological and social impacts can be immense.^7,8^ People in chronic pain often receive no clear medical diagnosis, making managers less likely to offer sympathy or accommodation^9^ and increasing the chances that these people will continue to work through pain and/or feel pressured into returning to work prematurely.^10^ In turn, this can result in increased job stress, high physical demands, job dissatisfaction, an unsupportive workplace, and more subsequent days off.^11^ Individuals with chronic pain may feel guilty, frustrated, and even a burden to colleagues, leading to a detrimental effect on their own mental health and a decline in morale across the workplace as colleagues become frustrated with having to pick up the workload as productivity levels wane due to both absenteeism and presenteeism.^12,13^

Though presenteeism has clear psychosocial and financial costs, these costs remain difficult to quantify. We are interested in exploring some of the indirect psychosocial costs that go above and beyond monetary loss which constitute a broader definition of presenteeism. This study aims to specify how chronic pain affects individuals within the workplace by examining psychosocial variables in a large sample of individuals in the European workforce.

## Method

### European Working Conditions Survey—Participants

Data were taken from the sixth and most recent European Working Conditions Survey (EWCS) collated in 2015 (see Data availability section).^14^ Ethics approval for the EWCS was provided by INRA EUROPE and participants gave informed consent. Analysis conducted for this article was performed on a fully anonymized and publicly available version of these data. Further information on how data  were collected can be found in  ‘Data availability section’.^14^

In total, 43,850 participants were interviewed face-to-face across 35 European countries. These included 28 European Union Member States, plus Albania, Turkey, Switzerland, the Former Yugoslav Republic of Macedonia, Montenegro, Norway, and Serbia. In most countries, the target sample size was 1000. To reflect the larger workforce in larger countries, the target was increased to 1200 in Poland, 1300 in Spain, 1400 in Italy, 1500 in France, 1600 in the UK, and 2000 in Germany and Turkey. Countries were also offered the opportunity to top up their sample. This offer was taken up by Belgium, Slovenia, and Spain, which led to sample sizes of 2500, 1600, and 3300 respectively.

Participants chosen were a random sample of people in employment, representative of the working population in each European Union country. This population included all active employed and self-employed persons aged 15 years and over (16 or older in Bulgaria, Norway, Spain, and the UK). People were classified as employed if they had worked for pay or profit for at least an hour in the week preceding the interview (International Labor Organization definition)^15^ or if they were not working but had jobs from which they were temporarily absent. All retirees, unemployed people, and homemakers were excluded. Non-Europeans were included on the condition that they were interviewed in the respective language of the country in which they worked.

A multistage, stratified, random sample of the working population was taken in each country to deliver a clustered sample. Depending on the availability of high-quality registers, sampling was carried out using individual-level, household-level, and address-level registers or through enumeration using a random walk approach. Country-level samples were stratified by region and degree of urbanization. In each stratum, primary sampling units were randomly selected proportional to size. Subsequently, a random sample of households was drawn in each primary sampling unit. Finally, unless individual-level registers were used, in each household the selected respondent was the person whose birthday would arrive next.

The survey consisted of a questionnaire that was administered in a face-to-face interview. The respondents were interviewed at home and the questionnaire consisted of 106 multiple-part questions that covered information on types of contracts, various health outcomes, and several aspects of working conditions, including the physical environment, psychosocial working conditions, workstation design, working hours, work organization, and social support at work. Only questions that imposed a potential psychosocial or cognitive impact to an individual within the workplace were considered for analysis.

Pain and non-pain samples were selected from the original EWCS 2015 data. Although there were no direct questions about chronic pain, we inferred pain status from a question on chronic health conditions, doing our utmost to ensure that our chronic pain group consisted only of individuals with pain as their primary chronic health issue. Participants who indicated that they had one or more of the following over the past 12 months: headaches; backache; muscular pains in shoulders, neck, and/or upper limbs (arms, elbows, wrists, hands, etc.); or muscular pains in lower limbs (hips, legs, knees, feet etc.) and who indicated that they had an illness/health problem that has persisted for longer than 6 months were classified as having pain. To minimize the number of participants whose primary persistent health problem was not pain, we excluded individuals who endorsed other illnesses or health problems such as hearing problems, skin problems, injuries, or any “others” in the past 12 months. We did not exclude individuals with anxiety or fatigue, given their high levels of comorbidity with pain. To reduce the possibility that findings were driven by the inclusion of individuals with anxiety and chronic fatigue as their primary chronic health issue, a parallel analysis was carried out excluding these individuals.

The non-pain sample was identified as participants who reported no illness or health problems over the last 12 months and no persistent health problems lasting over 6 months. The total sample that was deducted from the EWCS 2015 original data set (43,850 participants) suitable for analysis was 18,022. This sample consisted of 4254 respondents with pain and 13,768 without pain.

### Measures

The first step in analyzing the data was to recode relevant questions such that 1 on a Likert scale indicated the most positive response for each question, allowing for directional interpretations in later analyses. Secondly, to manage the large number of variables and ensure that items tapping the same latent construct were grouped together, questions related to workplace costs (46 items in total) were selected for principal components analysis (PCA). PCA was chosen because the primary objective was to reduce a large set of variables to a smaller, more interpretable set. Once these items underwent a varimax-rotated PCA, related items could be consolidated into suitable psychosocial factors for a more coherent analysis. A scree test was used to limit the number of components. This was done by ranking eigenvalues of the components’ eigenvectors derived from the variance–covariance matrix. Psychosocial factors with eigenvalues greater than 1 were regarded as significant for this study. Each varimax-rotated factor is comprised of items that yield a high covariance. Varimax-rotated factor loadings below 0.4 covariance were excluded from the factor. If factor scores overlapped (i.e., an item scored >0.4) for more than one factor; the lower factor score was disregarded. Internal consistency for each of the factor scores were examined using Cronbach’s alpha. Participants with missing data for our variables of interest were discarded from all analyses. The primary reason for missing data was the exclusion of self-employed individuals from many of the psychosocial variables examined. This ensured that our sample consisted only of people who were employed and paid a salary within an agency, ensuring that a hierarchical management structure was part of the social context. Following a chi-square test to assess age and sex differences between the pain and non-pain samples (see Results), the two samples were matched by age and sex, leaving a pain sample of 2384 and a non-pain sample of 2263.

To obtain odds ratios and assess the contribution of pain to differences in our workplace variables, we ran a binary logistic regression for each factor. Tests were Bonferroni-corrected to account for multiple comparisons. Psychosocial factors that survived Bonferroni correction were put into a one-way between-subjects multivariate analysis of covariance to determine whether the factors were significant as part of a model (i.e., did not explain variance already accounted for by other factors).

To confirm previous findings that pain is associated with higher levels of absenteeism, a *t* test was carried out to assess whether individuals with pain missed more working days than individuals without pain. Analysis of variance (ANOVA) was conducted to investigate whether any observed differences in psychosocial variables were a function of individuals with pain having different job characteristics (e.g., part-time/full-time work, hours worked, carrying or moving heavy loads, working with computers, sitting, dealing with clients, level of education, etc.). A parallel logistic regression was conducted to include substantive job characteristics as additional covariates. All statistical analyses were carried out using SPSS 25 (IBM Corp^14^).

## Results

### Participant Characteristics

There was a relatively even distribution of men (5191) and women (4865) across the whole sample, with a high preponderance of women in the pain group. Females comprised 57.4% of the pain group, significantly greater than the proportion in the non-pain group 45.5%, χ^2^(1, *N* = 10,056) = 102.87, *P* < 0.001. On average, the mean age of the pain sample was significantly higher than that of the non-pain sample, χ^2^(66, *N* = 10,021) = 646.70, *P* < 0.001 (Table 1). These findings are consistent with previous findings that females and older populations are more likely to live with chronic pain.^14,15^ To ensure that age and sex were not confounders, case–control matching was used to create a new data set that matched the pain and non-pain sample by age and sex, leaving a pain sample of 2384 and a non-pain sample of 2263. ANOVA showed that age, *F*(1,4637) = 2.79, *P* = 0.095, and sex, *F*(1,4644) = 0.11, *P* = 0.736, did not significantly differ between the pain and non-pain samples.

**Table 1.** Descriptive statistics for age and sex in pain vs. non-pain populations

	Males	Females	Missing	Total	Mean age (*n* = 10,021)^a^
Pain	1014	1369	1	2384	46.35 (*n* = 2380)
Non-pain	4177	3496	3	7676	39.62 (*n* = 7641)
All	5191	4865	4	10,060	41.22

**^a^**Age has lower total n as some people not give their age.

Six job characteristics appeared to be significantly associated with the pain sample. These were part-time work, fewer hours worked, carrying or moving heavy loads more frequently, sitting more frequently, dealing with people outside the workplace more frequently, and a lower level of education (see Table 2). However, the effect sizes for all but one of these factors were small. The job characteristic most substantively found in the pain group was carrying or moving heavy loads, *F*(1, 4,642) = 159.00, *P* < 0.001.

**Table 2.** Descriptive statistics for job characteristics in pain vs. non-pain groups

Job Characteristics	Total	Pain group	Non-pain group				
	Mean (SD)	Mean (SD)	Mean (SD)	*df*	*F*	Sig.	η^2^
Part-time or full-time (low score = more likely to work part-time)	1.79 (.41)	1.78 (0.41)	1.80 (0.40)	4,462	3.44	0.064	0.001
Hours worked per week	36.55 (10.47)	36.7 (11.08)	36.4 (9.79)	4,601	.931	0.335	<0.001
Carrying or moving heavy loads (low score = more frequently)	5.86 (1.66)	5.56 (1.84)	6.17 (1.38)	4,642	159.00	<0.001	0.033
Sitting (low score = more frequently)	4.25 (2.17)	4.22 (2.17)	4.28 (2.17)	4,642	.89	0.346	<0.001
Dealing directly with people who are not employees at your workplace (low score = more frequently)	3.85 (2.41)	3.70 (2.39)	4.00 (2.42)	4,638	17.84	<0.001	0.004
Working with computers, laptops, smartphones etc. (low score = more frequently)	4.17 (2.41)	4.15 (2.44)	4.19 (2.37)	4,641	.29	0.591	<0.001
Level of education (low score = lower level of education)	6.79 (2.99)	6.65 (3.02)	6.95 (2.96)	4,312	11.25	0.001	0.003
Is your household able to make ends meet? (low score = very easily)	3.12 (1.33)	3.27 (1.35)	2.96 (1.20)	4,627	70.57	<0.001	0.015

### Principal Components Analysis

PCA was carried out across the 46 items that were selected from the EWCS based on their relevance to psychosocial association to quality of life and workplace adjustment. The varimax-rotated factor loadings (items) were then inspected for conceptual coherence, to ensure that the eventual factor solution yielded interpretable psychosocial factors (Table 3). One item did not load on to any psychosocial factors (89h: If I were to lose or quit my current job, it would be easy for me to find a job of similar salary). The table also shows the variance explained by each factor.

**Table 3.** PCA for factor scores with alpha reliability

	Varimax-rotated factor scores	Rotated variance explained (%)	Alpha reliability for factor scores
1. Supervisor support		22.52	0.90
Q63f: Your immediate boss encourages and supports your development	0.80		
Q63e: Your immediate boss provides useful feedback on your work	0.81		
Q63b: Your immediate boss gives you praise and recognition when you do a good job	0.78		
Q63d: Your immediate boss is helpful in getting the job done	0.76		
Q63c: Your immediate boss is successful in getting people to work together	0.76		
Q63a: Your immediate boss respects you as a person	0.63		
Q70a: Employees are appreciated when they have done a good job	0.54		
2. Job responsibility		6.95	0.81
Q61n: You can influence decisions that are important for your work	0.76		
Q61d: You are involved in improving the work organization or work processes of your department or organization	0.71		
Q61i: You are able to apply your own ideas in your work	0.71		
Q61e: You have a say in the choice of your work colleagues	0.68		
Q61c: You are consulted before objectives are set for your work	0.66		
Q61f: You can take a break when you wish	0.57		
3. Team cohesion		5.16	0.84
Q70f: In general employees trust management	0.52		
Q70c: Conflicts are resolved in a fair way	0.57		
Q70d: Work is distributed fairly	0.59		
Q70b: Management trusts the employees to do their work well	0.58		
Q70e: There is good cooperation between you and your colleagues?	0.72		
Q89d: I generally get on well with my work colleagues	0.59		
Q61a: Your colleagues help and support you	0.45		
4. Discrimination		4.88	0.69
Q72c: Over the past 12 months at work, have you been subjected to any of the following: Discrimination linked to nationality?	0.67		
Q72b: Over the past 12 months at work, have you been subjected to any of the following: Discrimination linked to race, ethnic background, or color?	0.73		
Q72e: Over the past 12 months at work, have you been subjected to any of the following: Discrimination linked to religion?	0.68		
Q72g: Over the past 12 months at work, have you been subjected to any of the following: Discrimination linked to sexual orientation?	0.65		
Q72f: Over the past 12 months at work, have you been subjected to any of the following: Discrimination linked to disability?	0.56		
Q72d: Over the past 12 months at work, have you been subjected to any of the following: Discrimination on the basis of your sex?	0.52		
Q72a: Over the past 12 months at work, have you been subjected to any of the following: Age discrimination?	0.43		
5. Threats/abuse in the workplace		4.41	0.74
Q80c: Over the last month, during the course of your work have you been subjected to any of the following: Threats?	0.79		
Q80a: Over the last month, during the course of your work have you been subjected to any of the following: Verbal abuse?	0.73		
Q80d: Over the last month, during the course of your work have you been subjected to any of the following: Humiliating behaviors?	0.71		
Q81c: Over the last month, during the course of your work have you been subjected to any of the following: Bullying/harassment?	0.60		
Q81a: Over the last month, during the course of your work have you been subjected to any of the following: Physical violence?	0.60		
6. Perceived competence		3.04	0.74
Q61j: You have the feeling of doing useful work	0.72		
Q61h: Your job gives the feeling of work well done	0.71		
Q61k: You know what is expected of you at work	0.69		
Q61g: You have enough time to get the job done	0.51		
7. Job reward		2.87	0.80
Q89a: Considering all my efforts and achievements in my job, I feel I get paid appropriately	0.73		
Q89e: The organization I work for motivates me to give my best job performance	0.61		
Q89c: I receive the recognition I deserve for my work	0.62		
Q89b: My job offers good prospects for career advancement	0.65		
8. Sexual harassment		2.66	0.69
81b: Over the past 12 months, during the course of your work have you been subjected to any of the following: Sexual harassment?	0.86		
80b: Over the last month, during the course of your work have you been subjected to any of the following: Unwanted sexual attention?	0.84		
9. Workplace stress		2.32	0.47
Q61m: You experience stress in your workplace	0.75		
Q61o: Your job requires that you hide your feelings	0.75		
10. Job security		2.24	1.00
Q89g: I might lose my job in the next 6 months	0.66		

PCA yielded 10 psychosocial factors with eigenvalues above 1. Cronbach’s alpha scores below 0.6 suggest that the items within the factor may not be measuring the same underlying construct. This is seen under the work stress factor (α = 0.47). This factor was therefore discarded from the analysis.

To determine whether chronic pain had a significant association with any of these psychosocial factors, we compared pain and non-pain groups using a logistic regression analysis for each factor (see Table 4). For parallel analysis including heavy lifting as a covariate (see Supplementary Table 4a).

**Table 4.** Logistic regression showing impact of chronic pain on each psychosocial factor

Component/factor	Beta	SE	Sig.	OR	95% CI
Supervisor support	−0.26	0.03	<0.001	0.77	0.73–0.82
Job responsibility	−0.03	0.03	0.381	0.97	0.92–1.03
Team cohesion	−0.01	0.03	0.836	0.99	0.94–1.06
Discrimination	−0.25	0.04	<0.001	0.78	0.71–0.85
Threats/abuse	−0.39	0.04	<0.001	0.68	0.63–0.73
Job competency	0.10	0.03	0.005	1.1	1.03–1.18
Job reward	−0.50	0.03	<0.001	0.61	0.57–0.65
Sexual harassment	−0.03	0.03	0.395	0.97	0.91–1.04
Job security	0.13	0.03	<0.001	1.14	1.07–1.21

**Figure 1.**
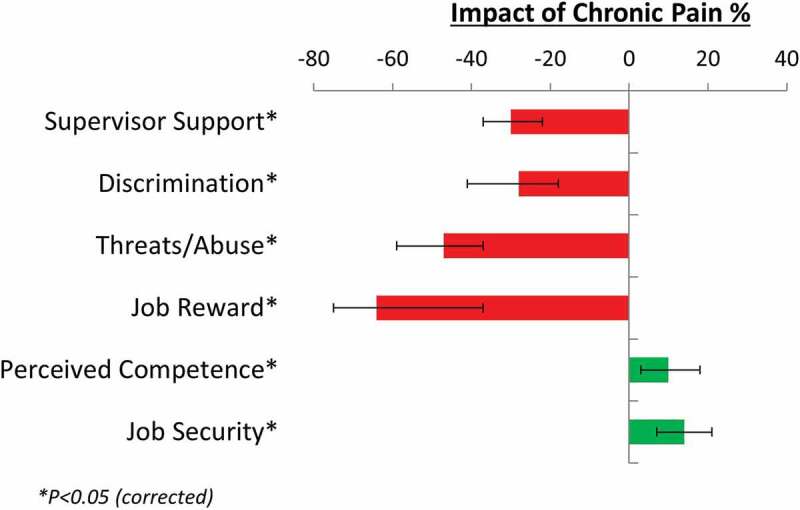
Effect of chronic pain on factor (odds ratios converted to percentages)

After Bonferroni correction (0.05/10, *P* < 0.005), three psychosocial factors were nonsignificant (job responsibility, team cohesion, and sexual harassment) and were not analyzed further. The other six psychosocial factors were significantly influenced by pain (see Figure 1). These same factors remained significant in our parallel analysis with individuals with anxiety and chronic fatigue removed (see Supplementary Table 1), indicating that these findings were likely not driven by those individuals. Similarly, the inclusion of heavy lifting as a covariate did not influence the findings (see Supplementary Table 2).

After inverting odds ratios for negative effects where appropriate, the significant findings of a logistic regression analysis for each factor was carried out because our core analyses showing that respondents with pain were 64% less likely to report their job as rewarding (odds ratio [OR] = 0.61, 1/0.61 = 1.64), 47% more likely to be subjected to threats and/or abuse in the workplace (OR = 0.68, 1/0.68 = 1.47), 30% more likely to report poor supervisor support (OR = 0.77, 1/0.77 = 1.30), and 28% more likely to perceive discrimination in the workplace (OR = 0.78, 1/0.78 = 1.28). On the other hand, respondents with pain were 10% more likely to report good job competency (OR = 1.10) and 14% more likely to report greater job security (OR = 1.14). There were no significant differences between the pain and non-pain respondents for job responsibility (OR = 0.97, *P* = 0.381), team cohesion (OR = 0.99, *P* = 0.836), and sexual harassment (OR = 0.97, *P* = 0.395) after Bonferroni correction. All odds ratios and 95% confidence intervals are provided in Table 4.

To examine the unique contribution of each of the significant psychosocial factors obtained from the univariate analyses, a multivariate ANOVA including all significant psychosocial factors was conducted (see Table 5). The model of psychosocial factors (supervisor support, discrimination, threats/abuse, perceived competence, job reward, and job security) was associated with chronic pain, *F*(6,4640) = 88.07, *P* < 0.001; Wilks’ Λ = 0.898, partial η^2^ = 0.102). All psychosocial factors remained significant within the model.

**Table 5.** Multivariate ANOVA comparing pain vs non-pain populations for each factor

	Group						
	Pain group		Non-pain group				
Factor	*M*	SD	*M*	SD	*F*	*P*	Partial η^2^
Supervisor support	0.22	1.18	−0.03	0.92	63.64	<0.001	0.014
Discrimination	0.11	1.27	−0.07	0.66	36.51	<0.001	0.008
Threats/abuse	0.28	1.38	−0.098	0.77	131.34	<0.001	0.027
Job competency	−0.16	0.94	−0.10	0.93	4.90	0.027	0.001
Job reward	0.34	1.05	−0.11	0.95	235.48	<0.001	0.048
Job security	−0.12	1.17	0.00	1.06	14.95	<0.001	0.003

Out of the total sample of 4647 who completed the selected questions, 4317 went on to answer another question about the number of working days they missed over the past 12 months. An exploratory *t* test was carried out to compare working days missed for the pain vs. non-pain respondents. People with pain missed three times as many days (mean = 13.69, SD = 28.96) of work per year than non-pain respondents (mean = 4.36, SD = 12.88), which was significant, *t*(4,315) = 13.44, *P* < 0.001, D = 0.40.

## Discussion

The study sheds light on some of the hidden costs of pain above and beyond absence from work and medical costs. Consistent with previous studies of absenteeism,^16,17^ individuals with pain missed three times as many days as individuals without pain. To assess the association of pain when individuals do show up for work (presenteeism), we compared individuals with and without pain on ten psychosocial factors: supervisor support, job responsibility, team cohesion, discrimination, threats/abuse, job competency, job reward, sexual harassment, stress and job security. Respondents with pain reported less reward for their efforts, poorer supervisor support, and higher rates of threats and abuse. Identifying these key aspects of presenteeism helps us to better understand the overall cost of chronic pain.

These findings are consistent with previous literature demonstrating the association between pain and poor relationships with managers in the workplace,^18^ discrimination,^19^ threats/abuse,^15,20^ greater stress levels,^21^ and feelings of underappreciation/low reward.^22^ Moreover, Nixon et al. conducted a meta-analysis across 79 studies and found that workplace stressors such as organizational constraints, interpersonal conflict, role conflict, role ambiguity, and lack of control were all related to physical symptoms.^23^ Psychosocial factors such as these have a negative effect on productivity in the workplace,^24–27^ with presenteeism often leading to burnout.^28–30^ One explanation for the feelings of discrimination/abuse, poorer supervisor relations, stress, and underappreciation in this study may be owing to respondents’ appraisals of employer concerns about productivity among respondents with pain.

In this study two considerable psychosocial stressors—discrimination and threats/abuse—were associated with pain. Sabbath et al. found similar results in a survey of 1497 care workers. They found that injury prevalence was associated with being yelled at, experiencing hostile/offensive gestures, and being sworn at.^20^ There is an increasing body of evidence that suggests that bullying and hostile work environments are associated with poorer health.^15,31–33^ Because these studies are cross-sectional, it is difficult to establish the directionality of the relationship between pain and social stress in the workplace, let alone the question of causality. The default presumption would be that pain is causing interpersonal issues, because pain regularly leads to socially detrimental behaviors such as frustration and depression.^34,35^ On the other hand, Nixon et al.’s meta-analyses included seven longitudinal studies indicating that workplace stressors may precede chronic pain development.^23^ It is also the case that such stress could exacerbate and maintain pain.^36–38^ It is possible that the relationship between pain and stress creates a negative feedback loop where pain causes interpersonal issues and is in turn exacerbated by those issues. Because the present study was also cross-sectional, this issue of causality between chronic pain and psychosocial factors applies, though it is likely that the two exacerbate each other.

Another notable finding of this study is the negative association of chronic pain with employee–supervisor relations. Studies have found that managers have limited awareness of employee pain and it is rarely openly discussed.^39^ Managers habitually do not consider pain among employees a problem,^9^ which is surprising given that a Danish study found that over 70% of the employees in a workforce had attended work despite experiencing considerable pain or sickness during the past year.^40^ One explanation that would reconcile these opposing points is that managers may have difficulty distinguishing chronic pain disorders from common day-to-day pain problems that most people experience at some point in their lives. This may be particularly important because studies have shown that management behavior can significantly influence how an employee handles pain at work.^41–43^ The Healthy Workplace Campaign was introduced to reduce workplace bullying in the United States, holding the employer accountable for an abusive work environment, which encourages employers to actively reduce hostility within the workplace.^44^ Similar campaigns may hold the key to reducing the negative psychosocial association with chronic pain in the workplace.^33,45,46^ The requirement for public and occupational health strategies to reduce and manage chronic pain is becoming increasingly important as the workforce is continuing to grow older and becoming more susceptible to health issues.^47,48^

Our study shows the negative association between pain and psychosocial factors in the workplace. The incorporation of campaigns that promote a work culture for handling pain could be of great benefit, helping to accommodate employees who struggle with pain and provide opportunities for trustful communication concerning employee health and possibilities for workplace adjustment. If campaigns can focus on creating a friendly environment that aids people in pain, it may be possible to address some of the psychosocial issues identified in this study. There is much literature that endorses the value of such strategies.^41,49–52^ For instance, employers report several advantages of accommodating employees with chronic health problems, with social inclusion having a positive effect on both productivity levels and employee health.^53^ Similarly, good leadership and a friendly social environment have been linked with enhanced productivity levels.^26,54^

A counterintuitive finding of the study was that people with pain reported that they were more competent and had greater job security than people without pain. A possible explanation for high perceived job security is that people with pain who were regularly absent and had strained relationships with their colleagues/supervisors may have read into their working rights more thoroughly and became more aware of the rules surrounding job security. The finding that people with pain perceived greater competence in their work suggests that people with pain did not feel that pain affected their job performance. An intriguing area for a follow-up investigation would be testing the possibility that people with pain are confident in their own abilities but feel a sense of injustice because they feel ostracized by colleagues and underappreciated for their efforts.

A limitation of the study was that little direct information about pain conditions was collected in the EWCS. As such, the presence of chronic pain was inferred from participants reporting they had been diagnosed with a health condition related to pain in the past 12 months and which had lasted more than 6 months. To mitigate the possibility that the observed workplace issues were due to a health condition other than pain, we excluded individuals who endorsed chronic health problems other than pain. Due to the high overlap between chronic pain and fatigue and anxiety symptoms, our primary analysis retained these individuals if they also endorsed pain symptoms, but we ran a parallel analysis without these individuals to confirm that our findings were not driven by either the inclusion or exclusion of individuals with comorbid anxiety or fatigue. Despite these steps, we cannot exclude the presence of some false positives in our sample. Moreover, there was no information on graded levels of pain intensity within the pain sample, and pain severity may ultimately be the most important factor in presenteeism and decreased productivity levels.^55^ An experimental paradigm designed for purpose would allow for more reliable data and greater accuracy. In addition, the data are retrospective and were obtained at the same time, so the temporal relationship between pain and psychosocial factors is unknown and we are therefore unable to determine causality between pain and psychosocial stressors. Moreover, the findings are subject to self-reported questionnaires, which may have led to people with pain reporting greater hardship within the workplace and social desirability bias leading to reports of greater competence at their job.

Though we have noted the limitations of our selection criteria for chronic pain, it is noteworthy that more than half of the workers with pain were women and the mean age for workers with pain was older than that of workers without pain. As the workforce is aging and people are working later into their life, workers with chronic pain will likely increase. If these findings are further verified, they suggest that employers will face greater psychosocial issues among the workforce related to chronic pain in the future and further social disparity may occur between individuals with pain and their coworkers if this issue is not addressed. As outlined above, campaigns such as the Healthy Workplace Campaign have been launched in the United States to successfully combat such issues.

This study detailed some of the psychosocial factors that should be considered when evaluating the costs of presenteeism. Some studies suggest that the costs of presenteeism may be considerably higher than those of absenteeism.^56,57, 58,59^ These comparisons, however, do not consider further potential losses if individuals with pain were to stay away from work. When considered in this light, presenteeism could be regarded as an act of organizational citizenship that garners praise. This view does not focus on productivity loss but on productivity gained compared to absenteeism.^5^ In terms of further study, these evaluations should be considered within the context of the impact on pain of working in a stressful psychosocial environment and whether such an environment might result in more missed work, higher medical costs, or even reduced productivity of nonaffected team members. This study attempted to identify some of the issues of presenteeism that may influence productivity levels within the workforce more broadly. Future studies should seek to quantify the effects of these issues on productivity.

## Supplementary Material

Supplemental Material

## Data Availability

https://www.eurofound.europa.eu/surveys/european-working-conditions-surveys-ewcs
