# Supplementary material for: Attending work with chronic pain is associated with higher levels of psychosocial stress
Source: Can J Pain. 2021 May 18;5(1):107–16. doi: 10.1080/24740527.2021.1889925 (PMC8210861; doi:10.1080/24740527.2021.1889925)
Supplement: Supplemental Material [file UCJP_A_1889925_SM7150.zip › Appendices_SupplementaryTable1 (V2) Matched.docx]

|  | Pain population with Anxiety and Fatigue as co-morbidities (n=2,384) | | | | | Pain Population without Anxiety and Fatigue as co-morbidities (n=863) | | | | |
| --- | --- | --- | --- | --- | --- | --- | --- | --- | --- | --- |
| Factor | Beta | SE | Sig. | OR | 95% CI | Beta | SE | Sig. | OR | 95% CI |
| Supervisor Support | -.26 | .03 | <.001 | .77 | .73-.82 | -0.14 | 0.04 | 0.001 | 0.87 | .81-.95 |
| Job Responsibility | -.03 | .03 | .381 | .97 | .92-.1.03 | 0.04 | 0.04 | 0.378 | 1.04 | .96-1.12 |
| Team Cohesion | -.01 | .03 | .836 | .99 | .94-.1.06 | 0.12 | 0.04 | 0.006 | 1.13 | 1.04-1.23 |
| Discrimination | -.25 | .04 | <.001 | .78 | .71-.85 | -0.15 | 0.05 | 0.002 | 0.86 | .78-.95 |
| Threats/ Abuse | -.39 | .04 | <.001 | .68 | .63-.73 | -0.22 | 0.05 | 0.000 | 0.80 | .74-.88 |
| Job Competency | .10 | .03 | .005 | 1.10 | 1.03-1.18 | 0.10 | 0.05 | 0.039 | 1.10 | 1.01-1.20 |
| Job Reward | -.50 | .03 | <.001 | .61 | .57-.65 | -0.31 | 0.04 | 0.000 | 0.73 | .78-.80 |
| Sexual Harassment | -.03 | .03 | .395 | .97 | .91-1.04 | 0.01 | 0.05 | 0.833 | 1.01 | .92-1.11 |
| Job Security | .13 | .03 | <.001 | 1.14 | 1.07-1.21 | 0.19 | 0.04 | 0.000 | 1.21 | 1.11-1.31 |

*Supplementary Table 1: Logistic Regression Comparison of pain population with and without co-morbidities against each factor (Age and Sex were used as co-variates).*
