# Supplementary material for: Attending work with chronic pain is associated with higher levels of psychosocial stress
Source: Can J Pain. 2021 May 18;5(1):107–16. doi: 10.1080/24740527.2021.1889925 (PMC8210861; doi:10.1080/24740527.2021.1889925)
Supplement: Supplemental Material [file UCJP_A_1889925_SM7150.zip › Appendices_SupplementaryTable2 V2 (Matched).docx]

|  | Logistic Regression excluding lifting heavy loads as Co-variates | | | | | Logistic Regression including lifting heavy loads as a Covariates | | | | |
| --- | --- | --- | --- | --- | --- | --- | --- | --- | --- | --- |
| Component/Factor | Beta | SE | Sig. | OR | 95% CI | Beta | SE | Sig. | OR | 95% CI |
| Supervisor Support | -.26 | .03 | <.001 | .77 | .73-.82 | -0.24 | 0.03 | <.001 | 0.79 | .74-.84 |
| Job Responsibility | -.03 | .03 | .381 | .97 | .92-.1.03 | -0.01 | 0.03 | 0.881 | 1.00 | .94-1.06 |
| Team Cohesion | -.01 | .03 | .836 | .99 | .94-.1.06 | -0.01 | 0.03 | 0.771 | 0.99 | .93-1.05 |
| Discrimination | -.25 | .04 | <.001 | .78 | .71-.85 | -0.24 | 0.04 | <.001 | 0.79 | .73-.86 |
| Threats/Abuse | -.39 | .04 | <.001 | .68 | .63-.73 | -0.37 | 0.04 | <.001 | 0.69 | .64-.74 |
| Job Competency | .10 | .03 | .005 | 1.1 | 1.03-1.18 | 0.12 | 0.04 | <.001 | 1.13 | 1.06-1.21 |
| Job Reward | -.50 | .03 | <.001 | .61 | .57-.65 | -0.47 | 0.03 | <.001 | 0.63 | .59-.67 |
| Sexual Harassment | -.03 | .03 | .395 | .97 | .91-1.04 | -0.02 | 0.03 | 0.544 | 0.98 | .92-1.05 |
| Job Security | .13 | .03 | <.001 | 1.14 | 1.07-1.21 | 0.14 | 0.03 | <.001 | 1.15 | 1.16-1.25 |

*Supplementary Table 2- Logistic Regression showing impact of chronic pain on each factor (With heavy lifting as co-variate).*
